# Diffusion indices alteration in major white matter tracts of children with tic disorder using TRACULA

**DOI:** 10.1186/s11689-024-09558-5

**Published:** 2024-07-17

**Authors:** June Christoph Kang, SuHyuk Chi, Young Eun Mok, Jeong-Ahn Kim, So Hyun Kim, Moon Soo Lee

**Affiliations:** 1 Department of Brain and Cognitive Engineering, Korea University, Seoul, Republic of Korea; 2 Department of Psychiatry, Korea University Guro Hospital, Seoul, Republic of Korea; 3 Department of medical science, Soonchunhyang University, Chungnam, Republic of Korea; 4 School of psychology, Korea University, Seoul, Republic of Korea

## Abstract

**Background:**

Tic disorder is a neuropsychiatric disorder characterized by involuntary movements or vocalizations. Previous studies utilizing diffusion-weighted imaging to explore white-matter alterations in tic disorders have reported inconsistent results regarding the affected tracts. We aimed to address this gap by employing a novel tractography technique for more detailed analysis.

**Methods:**

We analyzed MRI data from 23 children with tic disorders and 23 healthy controls using TRActs Constrained by UnderLying Anatomy (TRACULA), an advanced automated probabilistic tractography method. We examined fractional anisotropy (FA), radial diffusivity (RD), axial diffusivity, and mean diffusivity in 42 specific significant white matter tracts.

**Results:**

Our findings revealed notable differences in the children with tic disorders compared to the control group. Specifically, there was a significant reduction in FA in the parietal part and splenium of the corpus callosum and the left corticospinal tract. Increased RD was observed in the temporal and splenium areas of the corpus callosum, the left corticospinal tract, and the left acoustic radiation. A higher mean diffusivity was also noted in the left middle longitudinal fasciculus. A significant correlation emerged between the severity of motor symptoms, measured by the Yale Global Tic Severity Scale, and FA in the parietal part of the corpus callosum, as well as RD in the left acoustic radiation.

**Conclusion:**

These results indicate a pattern of reduced interhemispheric connectivity in the corpus callosum, aligning with previous studies and novel findings in the diffusion indices changes in the left corticospinal tract, left acoustic radiation, and left middle longitudinal fasciculus. Tic disorders might involve structural abnormalities in key white matter tracts, offering new insights into their pathogenesis.

## Introduction

Tic disorders represent a complex spectrum of neuropsychiatric conditions, characterized by involuntary, rapid, and recurrent motor movements or vocalizations, primarily observed in pediatric populations. These disorders, often preceded by distinctive premonitory sensations, typically manifest between the ages of 4 and 6, marking a critical developmental window in child neurology [3, 21]. The epidemiological profile of tic disorders is varied, with transient tics reported in 10–20% of school-aged children [38], while a comprehensive international meta-analysis indicates a global prevalence of 2.99% for transient tic disorder and 0.77% for chronic tic disorder [19, 37]. These figures underscore the public health significance of these conditions and highlight the need for a deeper understanding of their pathophysiology. Theoretical models elucidating the nature of tic disorders have evolved considerably over the years. The inhibition model, which posits a dysfunction in the cortico-basal ganglia-thalamo-cortical circuits, suggests impaired automatic inhibition of motor actions, while maintaining volitional control, as central to the development of tics [15, 31, 44]. However, while this model provides insights into the motor aspects of tics, it offers limited explanation of the sensory phenomena associated with them, such as premonitory urges [32, 36]. In contrast, the dopaminergic model offers a broader perspective, linking tic phenomenology to aberrations in dopamine neurotransmission. This model suggests that increased phasic dopamine release is intricately associated with both the generation of premonitory urges and the manifestation of tic symptoms [6, 24]. Further supporting this model are pharmacological studies that demonstrate the efficacy of dopamine antagonists in mitigating tic severity [38]. Additionally, neurochemical imaging studies have shown alterations in dopaminergic pathways in patients with tic disorders, reinforcing this hypothesis [8].

Diffusion tensor imaging (DTI) reveals the structural connectivity of the brain by measuring the diffusion of water molecules restricted by cell membranes of white matter tissue. The two common ways of analyzing DTI data are Tract-based spatial statistics (TBSS) and tractography. TBSS is an automated approach that allows whole-brain analysis of white matter in a voxel-wise manner, whereas tractography reconstructs 3-dimensional trajectories of specific white matter tracts, allowing a more detailed analysis of specific subpopulations of particular tracts. TRActs Constrained by UnderLying Anatomy (TRACULA) is one of the probabilistic tractography methods that has the advantage of being sensitive to the alterations of the predefined major white matter tracts [23, 46].

Previous studies on tic disorders have observed structural alteration in white matter. Increased fractional anisotropy (FA) in cerebello-thalamo-cortical tracts with an inverse linear relationship to tic severity [43], abnormalities among components of the fronto-striato-thalamic connections [25], and decreased FA in most of the major tracts including the internal capsule and corpus callosum were reported [33]. A study on adolescents also showed decreased axial diffusivity in various tracts including the corpus callosum, corticospinal tract, cingulum, anterior thalamic radiation, and inferior longitudinal fasciculus [41]. Adults with tic disorders showed lower FA values in the corpus callosum, particularly in fiber tracts connecting the bilateral primary and supplementary motor cortices [27]. Another structural network analysis study found reduced connectivity in the right hemisphere. The study also reported a positive correlation between global efficiency and YGTSS [39].

The presence of white matter abnormalities seems to be prominent among individuals with tic disorder. Furthermore, these pathophysiologic changes appear to continue from childhood through adulthood. Symptoms peak at around the ages of 10 to 12 and usually decline after puberty [20]. A plausible hypothesis regarding this subject suggests that puberty involves changes in white-matter structures [30], with particular emphasis on the corpus callosum [34]. Current study conducted on the adolescent sample to reflect the possible effect of the puberty of tracts in Tic disorder.

Yet, the specific tracts associated with the initiation and severity of tic disorder remain ambiguous. Previous studies adopted TBSS [33, 41], or ROI seeded probablistic tractography, only focused on tracts of interest or ROI from TBSS analysis [25, 27, 41, 43]. Given the practical difficulties of recruiting a sufficient number of participants in Tic disorders, restricting significant results to regions survived after multiple comparisons in a whole brain TBSS runs the risk of reducing power. An anatomically informed tracts-based approach is one alternative to address the issue considering the voxel-based nature of TBSS. Currunt study investigated white matter alteration in tic disorder using a novel method that can subdivide the major tracts in more detail. By adopting the novel tractography method allowing a more granular analysis of major white matter tracts, our approach seeks to delineate the specific neural pathways implicated in the onset and progression of tic disorders in more detail. This investigation is poised to enhance our understanding of the neurobiological basis of Tic disorder and potentially inform targeted therapeutic interventions.

## Materials and methods

### Participants

Patients were recruited from the department of psychiatry of Korea University Guro Hospital. Healthy controls were recruited from local schools and kindergartens. A total of 23 children with tic symptoms (mean age 10.40 ± 2.84 years) and 23 controls (mean age 10.00 ± 1.94 years) participated in the study. All participants had IQ scores above 70 on examination as measured by the Korean version of the Wechsler Intelligence Scale for Children fourth edition (K-WISC-IV), were free of psychotropic medication for at least 3 weeks, and had no history of neurological disorders including head trauma, tumors, or seizures. The demographics of all participants are summarized in Table 1, and there are no significant difference in demographic variables between two groups.

**Table 1:** Demographic and clinical characteristics

Variables	TIC (*n* = 23)	HC (*n* = 23)	*p*-value
Age (years)	10.4 (2.84)	10.0 (1.94)	0.630
Gender (male/female)	18/5	14/9	0.200
IQ	96 (9.21)	100.3 (8.12)	0.100
YGTSS-Total	24.78 (7.64)	N/A	N/A
YGTSS-Motor	7.70 (2.48)	N/A	N/A
YGTSS-Vocal	4.48 (4.43)	N/A	N/A
YGTSS-Severity	12.61 (5.41)	N/A	N/A

N/A: not available

### Diagnosis and clinical assessments

The diagnosis of tic disorders was made through clinical interviews by child and adolescent psychiatrists based on the 5th edition of the Diagnostic and Statistical Manual of Mental Disorders (DSM-5). Tic severity was assessed using the Yale Global Tic Severity Scale (YGTSS) [22]. Patients were assessed for psychiatric comorbidities using the Korean version of the Kiddie-Schedule for Affective Disorders and Schizophrenia-Present and Lifetime Version (K-SADS-PL) [18]. All research procedures were approved by the Institutional Review Board (IRB) of the Korea University Guro Hospital. Written informed consent was obtained from the parents or legal guardians of each participant. A more detailed description of the study procedures has been described in other published studies (e.g., [5]).

### Brain imaging procedure

MRI data were collected at Korea University Guro Hospital using a Siemens 3 T Magnetom Prisma (SIEMENS Healthineers, Erlangen, Germany) with a 32 channel Head Coil. T1-weighted anatomical images were obtained using a T1-weighted magnetization-prepared rapid gradient-echo sequence (repetition time: 2,300 ms; echo time: 2.32 ms; inversion time: 900 ms; flip angle: 8°; field of view: 230 mm; voxel size: 0.9 mm isotropic; 208 slices; generalized auto-calibrating partially parallel acquisition acceleration factor: factor of two along the phase-encoding direction; received bandwidth per pixel: 200 Hz/pixel; echo spacing: 7.1 ms). Then, whole-brain diffusion-weighted images were obtained using a single-shot spin-echo echo-planar sequence with 64 gradient orientations (number of directions: 64; repetition time: 3,100 ms; echo time: 78 ms; flip angle: 90°; acquisition matrix: 112 × 112; field of view: 224 mm; voxel size: 1.0 × 1.0 × 2.0 mm; slice thickness, 2.0 mm; averages: 2). One image without motion probing gradient (*b* = 0 s/mm^2^) and 64 images with it (*b* = 1,000 s/mm^2^) were obtained for each subject.

### Data processing

Cortical reconstruction and volumetric segmentation were performed with Freesurfer 7.3.2 (http://surfer.nmr.mgh.harvard.edu/*).* Automated segmentation of gray matter and white matter boundaries results were reviewed using Freeview for quality control and manually corrected when necessary according to the guidelines [14, 45]. Processed anatomical data is used for accurate white-matter reconstruction. Freesurfer TRACULA (TRActs Constrained by UnderLying Anatomy) software was used to reconstruct 42 major white-matter pathways using global probabilistic approach [23]. TRACULA calculates the probability distributions of neural pathways by combining a simplified model of diffusion called the “ball-and-stick” model with existing anatomical knowledge [46]. After determining the estimated distributions of the 42 major tracts, TRACULA derives four diffusion metrics for each tract: (1) fractional anisotropy (FA), which represents the fraction of diffusion that is directionally constrained; (2) axial diffusivity (AD), corresponding to the main direction of diffusion; radial diffusivity (RD), measuring the extent of diffusion perpendicular to the main direction; and mean diffusivity (MD), which indicates the overall magnitude of diffusion in the tissue. According to quality control pipeline [13], the resulting major 42 tracts were visually inspected to prevent the partially reconstruct tracts. No reconstruction failure or partially reconstructed tracts were found upon inspection.

### Statistical analysis

First the analysis of covariance (ANCOVA) was conducted using averaged diffusivity scores of each reconstructed major tracts, while control the effect of age, biological sex, and IQ. Further analysis was conducted along the tracts shown significant difference between groups. For each diffusivity indices, we fit a general linear model (GLM) with the along-tract diffusivity value as the dependent variable, and diagnosis as independent variables, while same nuisance variables are controlled. The GLM analysis was performed with mri_glmfit for 1D data, then all the group analyses were corrected for multiple comparisons using Monte-Carlo Simulation with mri_glmfit-sim [12]. The cluster-forming threshold and the cluster-wise threshold for statistical significance were both set to *p* = 0.05, and 5,000 simulations were performed. Along with the p-value, effect sizes were estimated using Cohen’s *d*, and the Bayes Factor Bound (BFB) is calculated and reported for provide an approximate upper bound for Bayes Factor [1]. The correlation analysis was performed between the diffusion indices of significant tracts and YGTSS scores reflecting the severity of motor symptoms.

## Results

### The integrity of the white matter tracts

**Fig. 1:**
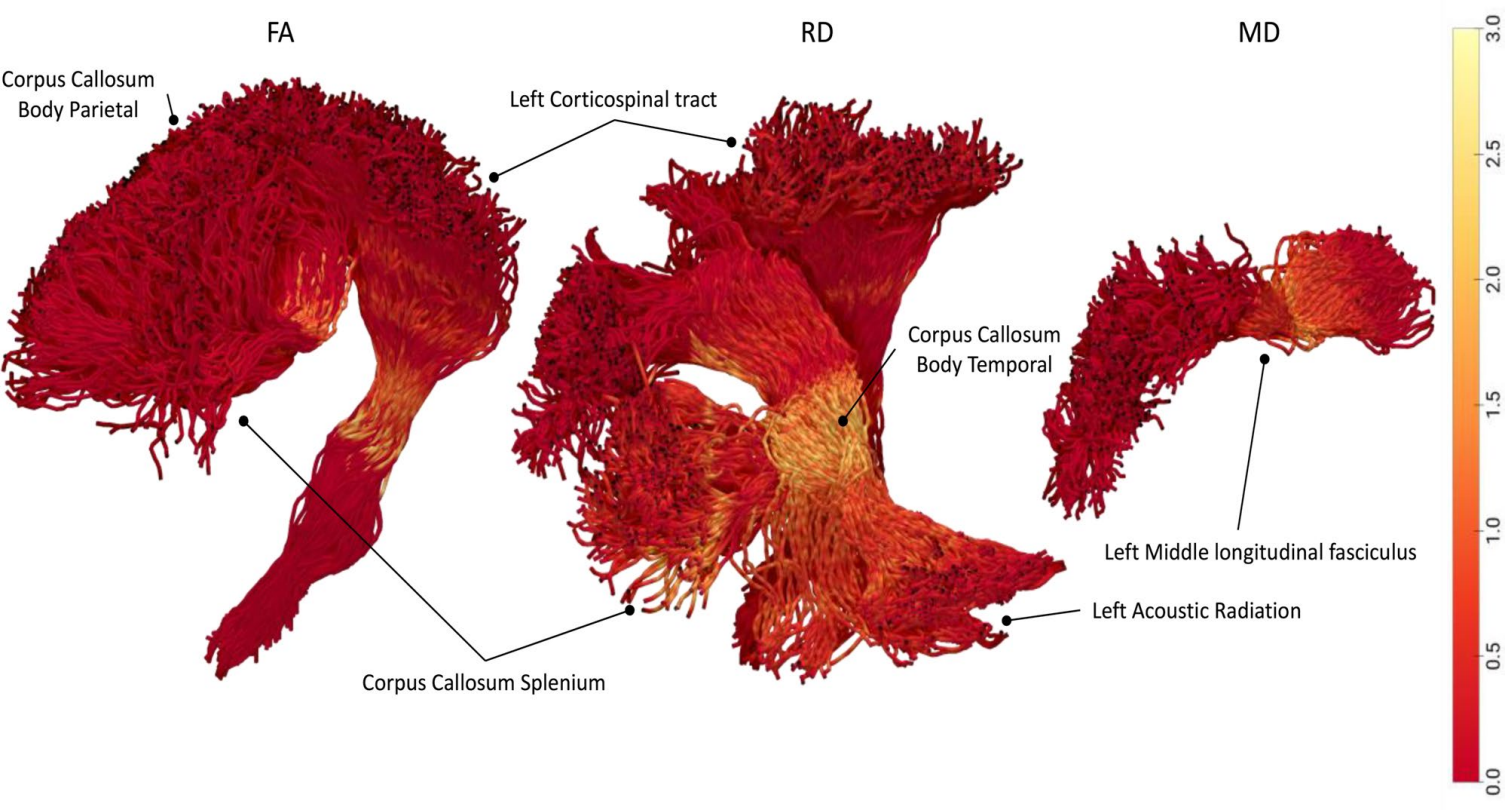
The major tracts shown significant difference between children with tic disorder and controls (FA : Fractional Anisotropy, RD: Radial Diffusivity, MD: Mean Diffusivity)

**Fig. 2:**
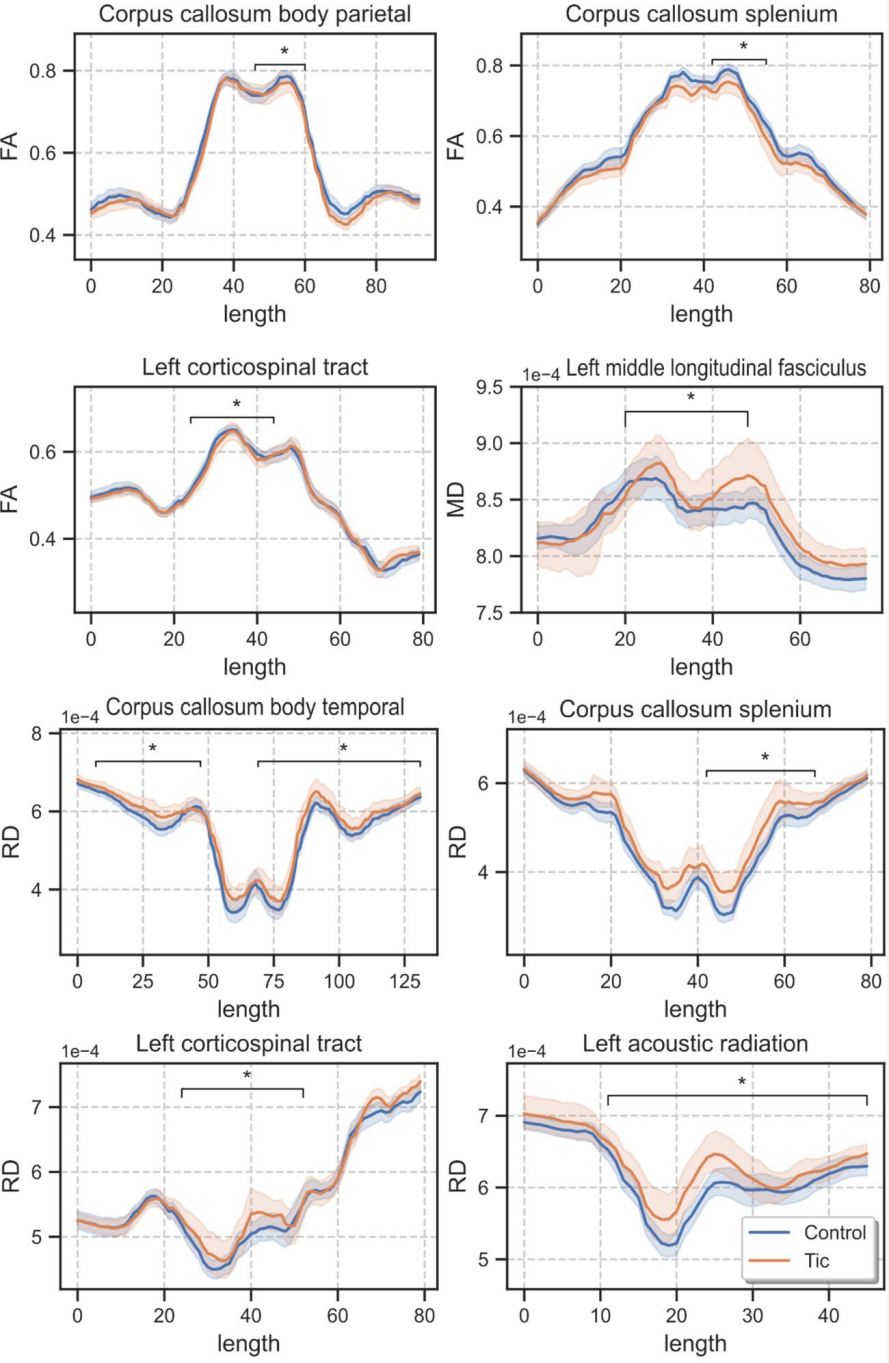
Along-the tract analysis of the tracts showing differences between children with and without tic disorder (FA : Fractional Anisotropy, RD: Radial Diffusivity, MD: Mean Diffusivity, length is the position along the tracts)

Relative to the control group, patients with tic disorder showed significantly lower FA values in the parietal part (*p* = 0.0010, *d* = 0.080, BFB = 53.26) and splenium of the corpus callosum (*p* = 0.0003, *d* = 0.387, BFB = 151.17), and in the left corticospinal tract (*p* = 0.0007, *d* = 0.794, BFB = 72.34). The patient group showed higher radial diffusivity (RD) values in the temporal part (*p* = 0.0011, *d* = 0.484, BFB = 49.09) and splenium of the corpus callosum (*p* = 0.0006, *d* = 0.209, BFB = 82.65), left corticospinal tract (*p* = 0.0009, *d* = 0.481, BFB = 58.28), and left acoustic radiation (*p* = 0.0010, *d* = 0.735, BFB = 53.26). The patient group also showed higher mean diffusivity (MD) values in the left middle longitudinal fasciculus (*p* = 0.0006, *d* = 0.178, BFB = 82.65, Table 2; Figs. 1 and 2).

**Table 2:** Diffusion indices of white matter tracts which showed significance difference between children with and without tic disorder

White matter tract	Index			B	*p*-value	Cohen’s d	Power	BFB
		Tic	HC					
Corpus Callosum Body Parietal	**FA**	0.505 (0.0253)	0.507 (0.0248)	2.999	0.0010	0.080	0.058	53.26
Corpus Callosum Splenium		0.560 (0.0427)	0.574 (0.0281)	3.485	0.0003	0.387	0.250	151.17
Left Corticospinal tract		0.640 (0.0519)	0.672 (0.0235)	3.132	0.0007	0.794	0.750	72.34
Corpus Callosum Body temporal	**RD**	0.000612 (0.0000473)	0.000592 (0.0000344)	-2.965	0.0011	0.484	0.362	49.09
Corpus Callosum Splenium		0.000560 (0.0000287)	0.000554 (0.0000288)	-3.192	0.0006	0.209	0.107	82.65
Left Corticospinal tract		0.000566 (0.0000441)	0.000547 (0.0000342)	-3.033	0.0009	0.481	0.358	58.28
Left Acoustic Radiation		0.000456 (0.0000684)	0.000417 (0.0000309)	-2.98	0.0010	0.735	0.684	53.26
Left Middle longitudinal fasciculus	**MD**	0.000843 (0.0000372)	0.000837 (0.0000298)	-3.211	0.0006	0.178	0.091	82.65

*FA: fractional anisotropy, RD: Radial Diffusivity, MD: Mean Diffusivity, BFB: Bayes Factor Bound

### Correlations between changes in the white matter and clinical scores

**Fig. 3:**
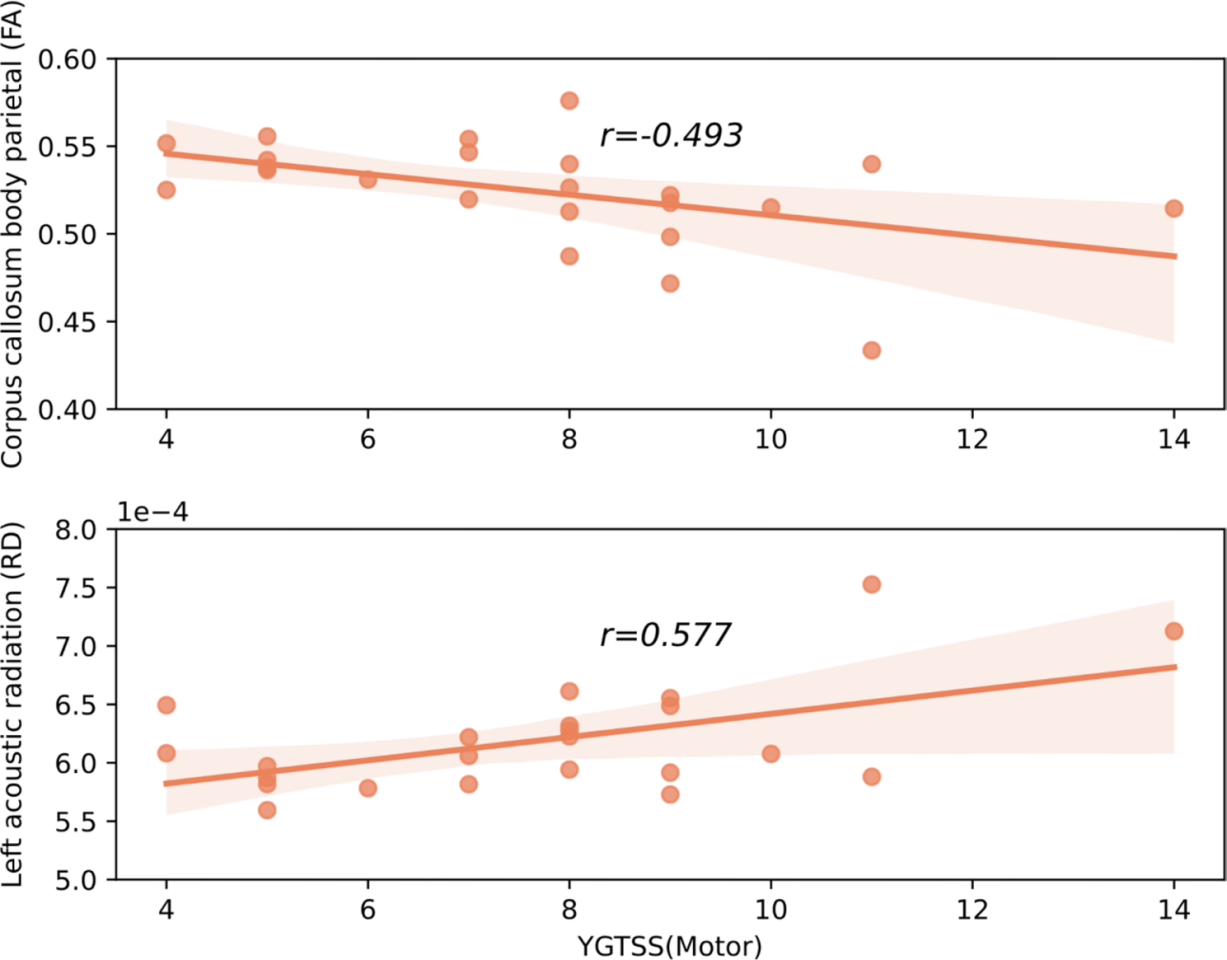
Correlation between YGTSS Motor symptom and diffusion indices of children with tic disorder (YGTSS: Yale Global Tic Severity Scale)

Significant correlations were observed between YTGSS motor scores and FA values of the parietal part of the corpus callosum (*r*= -0.493, *p* = 0.027) and RD values of the left acoustic radiation (*r* = 0.577, *p* = 0.008) while controlling for age, sex, and IQ (Fig. 3). No significant correlations were observed between YTGSS vocal scores and diffusivity indices.

## Discussion

Our study’s findings of altered white matter integrity in the corpus callosum, corticospinal tract, acoustic radiation, and middle longitudinal fasciculus in tic disorder patients underscores the neuroanatomical changes associated with the condition. Since the study conducted on the adolescent participants of mean age 10.40, the changes of above tracts might be the important markers of the onset and prognosis of the Tic disorder.

The corpus callosum, as the largest white matter structure in the brain, plays a pivotal role in coordinating motor functions and cognitive processes between the two hemispheres. Its impairment could lead to the disrupted synchronization of neuronal activity, which is crucial for the smooth execution of voluntary movements. The corpus callosum connects the two cerebral hemispheres and plays an important role in interhemispheric inhibition of voluntary movements [10]. It is suggested that the tract is also integral to the compensatory neuroregulatory system, which involves prefrontal regulation of motor activity within the cortico-striato-thalamo-cortical (CSTC) circuits in tic disorder [16, 27, 35]. FA reduction in the corpus callosum has been repeatedly observed among children and adults with tic disorders [2, 4, 16, 33, 35]. Children with tic disorder had lower FA values in all five subregions of the corpus callosum, and the result remained stable even after excluding patient with comorbid ADHD or OCD, as well as patients on medication [35]. The observed lower FA and higher RD in the corpus callosum, particularly, align with the hypothesis that tic disorders involve disruptions in interhemispheric communication. This is consistent with the theory that tics are a result of dysregulated networks involving both cortical and subcortical structures [42].

Furthermore, the alterations in the corticospinal tract observed in our study are particularly intriguing. The corticospinal tract is a pathway that originates in the primary motor cortex of the cerebrum and extends down through the brainstem and spinal cord. It plays a critical role in voluntary movement by transmitting signals from the motor cortex to the muscles. This tract is fundamental in the initiation and modulation of voluntary movements. The changes in FA and RD we noted could reflect a structural basis for the motor symptoms characteristic of tic disorders. Prior research has highlighted alteration of the corticospinal tract in tic disorder. Specifically, a decrease in FA and an increase in RD have been documented [33]. These alterations could be indicative of a broader disruption in the motor network, potentially contributing to the involuntary movements seen in these patients.

The increased RD in the left acoustic radiation found in our study is another novel finding. This tract originates in the medial geniculate nucleus (MGN) and curves anteriorly through the white matter within the transverse temporal gyrus to the primary auditory cortex [40]. The acoustic radiation, involved in auditory processing, has not been traditionally associated with motor function. However, our results suggest a possible link between auditory processing pathways and motor symptom severity in tic disorders. This could be reflective of the multisensory integration issues often reported in these patients with neurodevelopmental disorders [9]. The medial geniculate nucleus, from which the acoustic radiation originates, is known to have connections with various cortical and subcortical areas involved in motor control. Thus, alterations in this pathway could contribute to the complex sensorimotor integration challenges observed in tic disorders.

The elevated MD values in the left middle longitudinal fasciculus (MdLF) observed in our patient group also warrant attention. The MdLF is an associative longitudinal fiber connecting the superior temporal gyrus and the inferior parietal lobule, specifically the angular gyrus [26, 29]. The MdLF was believed to perform language-related functions. However, an intraoperative study found that electrostimulation of MdLF did not interfere with picture naming tasks, and its surgical removal did not result in any language deficits [7]. Current hypotheses suggested that the MdLF, particularly on the left hemisphere, plays a role in the associative learning of verbal-auditory stimuli [20]. The MdLF, implicated in language processing and associative learning, may have a more nuanced role in tic disorders than previously thought. The association between structural changes in the MdLF and tic disorders could reflect underlying disruptions in the neural networks involved in complex cognitive functions. This is in line with recent studies suggesting that tic disorders are not merely motor disorders but involve a broader spectrum of cognitive and sensory processing anomalies [28]. In addition, the elevated MD values in the left MdLF observed in our patient group can be related to previous studies interpreting tic disorder as a problem at the temporo-parietal junction [11].

Several limitations of this study should be noted. Although this research found several differences between children with and without tic disorders, the sample size of the study is relatively small for general neuroimaging studies. Therefore, the results of this study should be interpreted with caution, considering the effect size is small to moderate.

Despite these limitations, the current findings highlight the difference in major white matter tracts of the children with Tic disorder compare to typically developed controls. The finding is in line with the recent studies conceptualize the Tic disorder as the disrupted network function [17, 47].

## Conclusion

Taken together, our findings suggest that tic disorders are characterized by a complex pattern of white matter alterations, not limited to motor pathways but extending to sensory and cognitive processing networks. This aligns with the emerging perspective that tic disorders are a result of widespread neurodevelopmental anomalies affecting multiple brain networks. Future research should focus on longitudinal studies to understand the progression of these white matter changes and their relationship with clinical symptoms. Additionally, exploring the potential of these neuroimaging findings as biomarkers for early diagnosis and treatment response in tic disorders could be a promising avenue. The integration of neuroimaging with genetic and clinical data may also provide deeper insights into the etiology and pathophysiology of tic disorders, paving the way for more targeted therapeutic interventions.

## Data Availability

The datasets generated and analyzed during the current study are available from the corresponding author upon reasonable request.
